# Beyond physiotherapy and pharmacological treatment for fibromyalgia syndrome: tailored tACS as a new therapeutic tool

**DOI:** 10.1007/s00406-020-01214-y

**Published:** 2020-11-25

**Authors:** Laura Bernardi, Margherita Bertuccelli, Emanuela Formaggio, Maria Rubega, Gerardo Bosco, Elena Tenconi, Manuela Cattelan, Stefano Masiero, Alessandra Del Felice

**Affiliations:** 1 Department of Neuroscience, Section of Rehabilitation, University of Padova, via Giustiniani 3, 35128 Paduas, Italy; 2 Department of Neurosciencse and Padova Neuroscience Center, University of Padova, 35131 Padua, Italy; 3 Department of Biomedical Sciences, University of Padova, Via Marzolo 3, 35031 Padua, Italy; 4 Department of Neuroscience and Padova Neuroscience Center, Psychiatric Clinic, University of Padova, Via Giustiniani 3, 35128 Padua, Italy; 5 Department of Statistical Sciences, University of Padova, via C. Battisti 241, 35121 Padua, Italy

**Keywords:** Pain, Fibrofog, Non-invasive transcranial stimulation, Rehabilitation, Random noise stimulation (RNS)

## Abstract

Fibromyalgia syndrome (FMS) is a complex pain disorder, characterized by diffuse pain and cognitive disturbances. Abnormal cortical oscillatory activity may be a promising biomarker, encouraging non-invasive neurostimulation techniques as a treatment. We aimed to modulate abnormal slow cortical oscillations by delivering transcranial alternating current stimulation (tACS) and physiotherapy to reduce pain and cognitive symptoms. This was a double-blinded, randomized, crossover trial conducted between February and September 2018 at the Rehabilitation Unit of a teaching Hospital (NCT03221413). Participants were randomly assigned to tACS or random noise stimulation (RNS), 5 days/week for 2 weeks followed by ad hoc physiotherapy. Clinical and cognitive assessments were performed at *T*_0_ (baseline), *T*_1_ (after stimulation), *T*_2_ (1 month after stimulation). Electroencephalogram (EEG) spectral topographies recorded from 15 participants confirmed slow-rhythm prevalence and provided tACS tailored stimulation parameters and electrode sites. Following tACS, EEG alpha1 ([8–10] Hz) activity increased at *T*_1_ (*p* = 0.024) compared to RNS, pain symptoms assessed by Visual Analog Scale decreased at *T*_1_ (*T*_1_ vs *T*_0_ *p* = 0.010), self-reported cognitive skills and neuropsychological scores improved both at *T*_1_ and *T*_2_ (Patient-Reported Outcomes in Cognitive Impairment, *T*_0_–*T*_2_, *p* = 0.024; Everyday memory questionnaire, *T*_1_ compared to RNS, *p* = 0.012; Montréal Cognitive Assessment, *T*_0_ vs *T*_1_, *p* = 0.048 and *T*_0_ vs *T*_2_, *p* = 0.009; Trail Making Test B *T*_0_–*T*_2_, *p* = 0.034). Psychopathological scales and other neuropsychological scores (Trail Making Test-A; Total Phonemic Fluency; Hopkins Verbal Learning Test-Revised; Rey–Osterrieth Complex Figure) improved both after tACS and RNS but earlier improvements (*T*_1_) were registered only after tACS. These results support tACS coupled with physiotherapy in treating FMS cognitive symptoms, pain and subclinical psychopathology.

**Electronic supplementary material:**

The online version of this article (10.1007/s00406-020-01214-y) contains supplementary material, which is available to authorized users.

## Introduction

Fibromyalgia syndrome (FMS) is a complex chronic pain disorder, defined as widespread musculoskeletal pain in the absence of demonstrated tissue damage, and associated with symptoms ranging from affective disturbances, fatigue, and sleep alterations, to cognitive dysfunctions [1]. Cognitive difficulties are referred by 50–80% of people with FMS [2] and are ranked as the fifth most severe symptom [3]. “Fibrofog” was coined to describe the typical subjective experience of cognitive dysfunction in FMS, characterized among other, by concentration difficulties, forgetfulness, mental confusion and inability to multitask [4]. One of the most frequently reported cognitive dysfunctions in FMS is attention deficit [5]: people with FMS show poor performances in cognitive tests requiring to deal with distractors or any source of stimulus competition, such as divided attention, inhibition, set-shifting [6], working memory [7], semantic memory and speed of processing [8]. It is still unclear if these cognitive symptoms are primarily disease manifestation or a consequence of it. Many co-occurring symptoms in FMS such as depression, anxiety, sleep disturbances and pain perception may contribute and account for cognitive problems, even if no study found an unequivocal relation between these factors and the cognitive performance [4]. However, a relationship between pain intensity, affective and cognitive dysfunction has been postulated based on the overlap of brain areas involved in pain processing and cognition [9, 10]. This observation leads to the hypothesis that cognitive alterations in FMS arise because of resource competition with pain processing [6].

Physiotherapy is nowadays the most widely recognized and beneficial treatment for fibromyalgic pain symptoms. Literature reports effective pain and depression reduction associated with aerobic and strengthening exercises [11]. However, physiotherapy alone is not able to provide long-lasting effects involving other symptoms than pain.

Current research is focusing on biomarkers which may account for both pain processing alterations and cognitive fibrofog. Single-photon Emission Computed Tomography (SPECT) and Functional Magnetic Resonance Imaging (FMRI) revealed abnormal activation of thalamic nuclei, sensory cortex, anterior cingulate, insula and prefrontal cortices during pain processing in FMS [12, 13]. Electroencephalographic (EEG) studies show increased theta rhythm primarily localized in frontal brain regions and anterior cingulate cortex [14, 15], which are part of the thalamo-cortical circuit. On this ground, chronic pain referred by people with FMS has been interpreted as the result of a “thalamo-cortical dysrhythmia” [14] characterized by a shift of oscillatory frequencies in the thalamo-cortical circuits.

Neuromodulation techniques [non-invasive brain stimulation (NIBS): transcranial magnetic (TMS) and transcranial direct current stimulations (tDCS)] may modulate EEG frequency rhythms [16, 17] and shift cortical EEG generators [18, 19]. Up to now, NIBS has been mainly applied in FMS with the hypothesis of reducing the increased activity described over prefrontal and sensory cortices. TDCS was previously administered with this aim. By generating low-intensity sub-threshold electrical fields, tDCS is able to modify neuronal transmembrane potentials and in turn modulate cortical excitability by bringing the underlying neurons closer to their firing threshold [20, 21]. Repetitive TMS (rTMS) delivered over M1 was administered as well to reduce FMS pain symptomatology [22]. However, studies on the effect of rTMS and tDCS report a variable efficacy on amelioration of symptoms and quality of life in FMS [23, 24].

Focusing instead on abnormal oscillatory activity in FMS, EEG activity normalization may be considered a therapeutic target. No study has explored the effect of transcranial alternating current stimulation (tACS) for the treatment of this clinical population [25].

TACS is a non-invasive, handy technique which may modulate endogenous brain oscillations [18, 25] when administrated as an alternate, sinusoid current. It has been demonstrated that tACS is able to shift EEG rhythms in other thalamo-cortical dysrhythmias [26]. Unlike tDCS, tACS does not induce any polarization effect but can modulate the ongoing brain activity by forcing the membrane potential to oscillate away from its resting state towards hyper-polarized or depolarized state. This results in the so-called entrainment effect: increasing of neuronal firing time-locked to the frequency of stimulation [27].

We hypothesize that tACS delivered over the cortical area showing the greatest EEG alteration (i.e., higher slow rhythms power) may have beneficial effects on both pain and cognition by shifting EEG activity towards physiological frequencies. To test this hypothesis, we applied tACS as a primer for a specific rehabilitation program.

## Materials and methods

The randomized, double-blind, crossover design was approved by the ethics Committee of the teaching Hospital of Padova University, Italy, (protocol no. 3507/AO/15). Each participant, before taking part in the experiment, was informed about the study and provided written informed consent. The study was registered on ClinicalTrials.Gov (NCT03221413).

The present study reports preliminary data from participants with chronic pain. The original protocol planned inclusion of individuals with neuropathy; due to the principal investigator change of affiliation, access to this clinical population was no more possible and FMS was included, given that both FMS and neuropathic pain are characterized by increased slow thalamo-cortical oscillations in theta frequency band [14, 16]. This allowed the application of the same stimulation protocol. Clinical and neuropsychological tests were adjusted and tailored to FMS. To enhance research transparency, we used the recently updated CONSORT guidelines for cross-over trials [28] and the CONSORT checklist for crossover designs (see Supplementary material 1).

Primary outcome was reduction of the main FMS symptom, pain, measured with a visual analog scale (VAS) on a Likert scale from 0 to 10.

### Participants

Participants (*N* = 15, 2 males; age: mean $\pm$ standard deviation: $53.07\pm 4.18$ years) were recruited as volunteers by the Italian Association of FMS (AISF) and enrolled by a blinded researcher in charge of the study conduction (LB), who also assigned them to sequence of intervention. Inclusion criteria were: diagnosis according to the diagnostic criteria for fibromyalgic syndrome [29]; score higher than 3 at the Visual Analog Scale for pain (VAS); pain non-responsive to at least two analgesic drugs administered in adequate dose for at least 3 months; stable pharmacological treatment during the study. Exclusion criteria were: contraindications to neurostimulation (pregnancy, metal fragments/implants, epilepsy, previous skull fractures, pacemaker); comorbid psychiatric illnesses or substance abuse disorders; minor age. Scalp EEG control data from twenty-one healthy volunteers (9 males; age: 45.14 $\pm$ 14 years) were obtained [30] using the same EEG setting.

### EEG data acquisition and analysis

Five minutes of open-eyes resting-state EEG signal (32-channel system; BrainAmp 32MRplus, BrainProducts GmbH, Munich, Germany) were acquired at a sampling rate of 5 kHz with the reference between Fz/Cz and ground anterior to Fz positioned according to a 10/10 system, band-pass filtered at 0.1–1000 Hz and digitized.

The data were processed in Matlab (MathWorks, Natick, MA) using personalized scripts based on EEGLAB toolbox [31]. The EEG recordings were band-pass filtered from 1 to 30 Hz and down-sampled at 500 Hz. Visible artifacts (eyes movements, cardiac activity, and scalp muscle contraction) were removed using independent component analysis, and data were processed with a common average reference. Two-seconds EEG epochs (i.e., non-overlapping segments of 1000 samples) were extracted for each participant and a fast Fourier transform (FFT) was applied. The recordings were Hamming windowed to control for spectral leakage. Power spectral density (μV^2^/Hz) was estimated for all frequencies and the relative power (%) was computed by dividing the power of each frequency band (delta [1–4 Hz], theta [4.5–7.5 Hz], alpha1 [8–10 Hz], alpha2 [10.5–12.5 Hz], beta [13–30 Hz]) with the total power in the range 1–30 Hz.

A *z* test (*p* < 0.05) was used to compare each participant versus controls and a statistical map, defining the electrodes in which relative power value differs from those of the control group, was provided [32].

### Trial design, stimulation parameters and tACS treatment

**Fig. 1:**
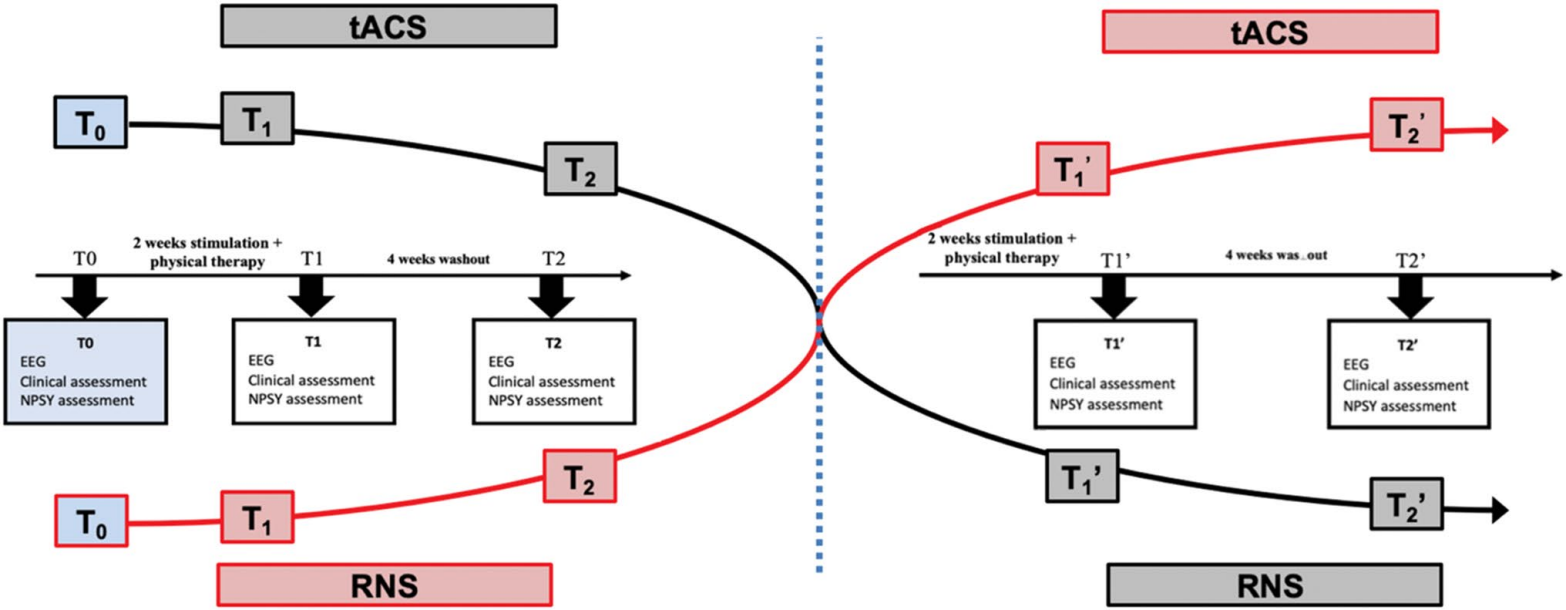
Schematic representation of the experimental design

Participants were randomly assigned with a computer-generated list (allocation ratio 1:1) to tACS or random noise stimulation (RNS as active sham). Each participant underwent 10 stimulation sessions lasting 30 min, 5 times a week, for two consecutive weeks, followed by 60 min of physical rehabilitative exercise. After a wash-out interval of 4 weeks from the conclusion of the first cycle, participants were crossed to the other group (Fig. 1). Participants were tested before program start (*T*_0_), at conclusion of each cycle [stimulation and physical rehabilitation (*T*_1_; *T*_1_′)], and after the 4-week wash-out interval (*T*_2_; *T*_2_′), with resting EEG, VAS, SF36, neuropsychological tests and questionnaires. *T*_0_ values were recorded only before the start of the whole experiment.

**Fig. 2:**
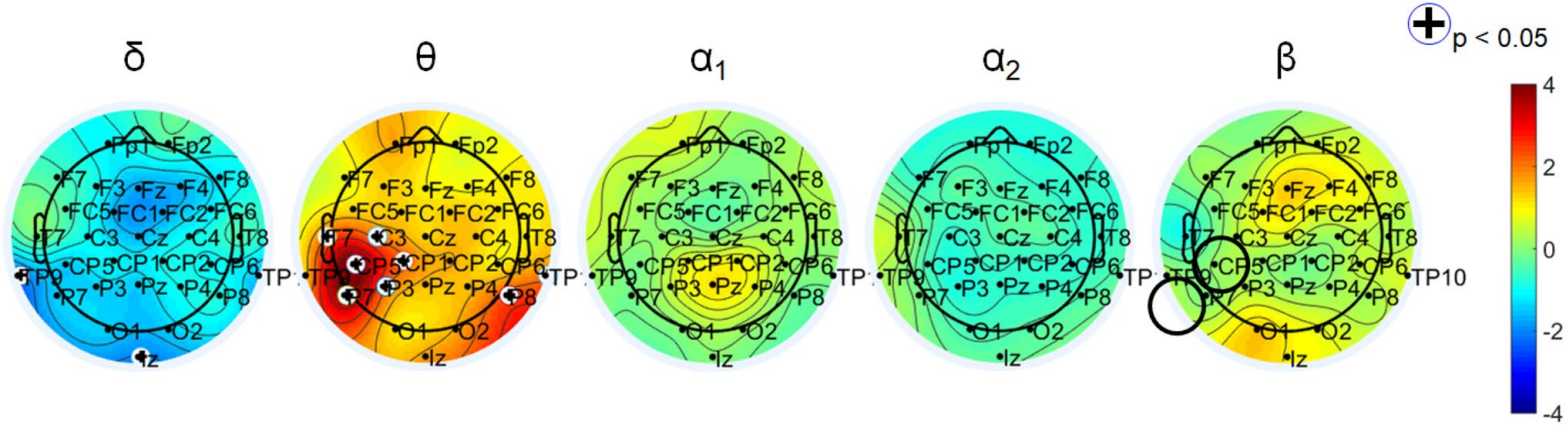
Statistical maps (*z* values) derived from one subject with FMS vs. control group. Participant n. 6 shows higher theta activity, compared to controls, over left motor area. She was stimulated with beta tACS over CP5 (black circles: anode over area of highest theta power, cathode over ipsilateral mastoid)

Baseline (before the whole protocol started) resting-state cerebral activity provided stimulation parameters (frequency and anode position on the scalp). EEG from participants was compared with those of a healthy control group to identify scalp areas in which a significant difference in frequency band spectral power was detected. We supported the hypothesis that a non-invasive stimulation though potentially different from the natural oscillations of the target brain region could modulate neuronal networks and shift oscillations into a physiological range. For this reason, we compared the power spectra of the participants with the one of the controls. Each participant was stimulated in the EEG frequency band that presented lower amplitudes compared to controls. Those showing higher slow frequencies (theta, delta, alpha1) spectral power were stimulated with beta-tACS at 30 Hz, while the ones showing higher fast frequencies (beta, alpha2) were stimulated with theta-tACS at 4 Hz. Figure 2 displays statistical maps derived from one participant showing higher theta activity over left motor area, compared to controls, and thus stimulated with beta-tACS over that cortical area. In case a subgroup should display a prevalence of faster frequencies (alpha2, beta), they were to be stimulated with slow tACS at 4 Hz.

Stimulation was applied by a battery driven external stimulator (BrainStim, E.M.S., Bologna, Italy) via two sponge electrodes (5 × 7 cm), with an intensity ranging from 1 to 2 mA. Anode was positioned for each subject over the scalp area showing highest power spectral difference; cathode over the ipsilateral mastoid. The RNS was an alternate current with random amplitude and frequency, respectively, in the intervals (1–2) mA and (0–100) Hz, with electrodes applied over the same sites as for real stimulation”. [17].

### Clinical assessment

The following clinical scales were administered to test pain and the self-reported health state:

1. Visual Analog Scale (VAS) [33]: a 10-point Likert scale, ranging from none to extreme amount of pain.
2. Short Form 36-item Health Survey (SF-36) [34]: the short form of a questionnaire inquiring participants’ health state. The 36 items are clustered into 8 domains: physical activity/functionality (10 items), limitations due to physical health (4 items), limitations due to emotional status (3 items), physic pain (2 items), general healthy sate perception (5 items), energy/fatigue (4 items), social activities (2 items), mental health (5 items) and one single question on the perceived changes in the health state.

### Neuropsychological and psychopathological assessment

To test cognitive, affective and psychopathological domains, we designed a battery which comprises the following standardized neuropsychological tests and questionnaires for:

1. Psychopathological self-assessment: Beck Depression Inventory-II (BDI-II) [35]; Brief Symptom Inventory (BSI) [36]; the State–Trait Anxiety Inventory [37].
2. Self-reported cognitive assessment: Patient-Reported Outcomes in Cognitive Impairment (PROCOG-P) [38]; Everyday Memory Questionnaire Revised (EMQ-R) [39].
3. Neuropsychological assessment: the Montréal Cognitive Assessment (MoCA) [40]; the Rey–Osterrieth Complex Figure Test [41]; the Digit Symbol-Coding (from the Wechsler Adult Intelligence Scale 4th edition [42]; the Hopkins Verbal Learning Test-Revised [43]; the Trail Making Tests A and B [44]; the Phonemic Verbal Fluency task [45]. For detailed description of each test, see Appendix A.

### Physiotherapy program

The European League Against Rheumatism (EULAR) guidelines have been used to design the physiotherapy program [46]. Physiotherapy program included 30 min of aerobic exercise followed by 15 min of muscular stretching and 15 min of breathing and guided relaxation techniques. Participants received 60-min physical rehabilitation at the end of each stimulation session, 5 times a week, for two consecutive weeks, both in the first and second experimental arms. See the online resources (Table T1) for the complete physiotherapy program.

### Statistical analysis

Region of interests (ROIs) were identified based on electrodes location: right frontal ROI: Fp2, F8, F4; right motor ROI: FC2, FC6, C4, Cp2; right parietal ROI: CP6, P8, P4. Contralateral ROIs were identified on the left hemisphere. EEG frequency power was calculated for each of these ROIs and used for subsequent analysis. Statistical analyses aimed at detecting differences in EEG frequencies, neuropsychological, psychopathological or clinical variables with respect to baseline and intervention. Each comparison was based on complete case analysis, so all available subjects were considered when their observations could be collected. Non-parametric methods were employed due to sample size. Spectral power of all EEG bands over different ROIs were compared to detect differences between the conclusion of the cycle (*T*_1_) and the end of the wash-out interval (*T*_2_) and between the condition of the subjects in each of those times (*T*_1_ and *T*_2_) and their baseline (*T*_0_) by means of the Wilcoxon signed-rank test for paired samples. EEG frequencies were compared also between tACS and RNS with a paired samples Wilcoxon signed-rank test. All changes in variables related to the neuropsychological and psychopathological assessments and to the clinical assessment collected at different time point were tested with the same scheme with a Wilcoxon signed-rank test, assuming the usual critical level 0.05. All statistical analyses were performed with the R statistical software [47].

### Test power calculation

Sample size was computed on the basis of a functional outcome which is not available for subjects who actually entered the study. For this reason, an analysis of the power of the test employed was performed. The primary outcome is the change in the VAS scale between the beginning of the study and the end of intervention. Since the Wilcoxon rank-sum test is a non-parametric test, the assumption of a standard parametric distribution for the data under the alternative hypothesis is avoided. A discrete distribution is assumed for the difference in VAS which ranges between − 1 and 6, with probabilities equal to those empirically observed, thus the median difference equals 1.5. Under such assumptions, a simulation study was performed in order to detect the power of the Wilcoxon signed-rank test. Based on 10,000 simulations, with the observed sample size, the power of the test is 85%.

## Results

Twenty-four participants were recruited. Enrolment flow diagram is reported in the online resources (Fig. F1). For demographic characteristics see Table 1. Seven participants did not meet inclusion criteria (2 had contraindication to tACS, 3 had a psychiatric disease, 2 had recently changed drug regimen). Of the remaining 17, 15 completed the first arm of the study (1 drop out, 1 change of drugs during trials), and 11 both study arms (1 change of drugs, 1 due to stroke, 2 dropouts for personal reasons).

**Table 1:** Demographic and clinical characteristics of included participants

Subject	Age (years)	Sex	Education (years)	Prevailing rhythm	Stimulation site	Stimulation frequency	Pharmakon
1	51	F	16	Theta	F3	30 Hz	Duloxetine (60 mg)
2	49	F	12	Theta	C3	30 Hz	Amitriptyline (10 mg ttx2)
3	51	F	13	Theta	Cp5	30 Hz	Hydroxychloroquine (200 mg)
4	56	F	13	Alpha 1	C4/Cp2	30 Hz	Tizanidine (4 mg)
5	55	F	16	Theta	C3/Cp5	30 Hz	Venlafaxine (75 mg)
6	49	F	9	Theta	Cz-Fz	30 Hz	Gabapentin (300 × 3), venlafaxine (75 mg)
7	55	F	8	Theta	Cp5	30 Hz	Alprazolam (0,25 mgx2), pregabalin (150mgx3), Trzodone (100 mg)
8	50	M	11	Alpha 2	Cz-Pz	4 Hz	Alprazolam (25mgx2), Amitriptyline (10 mg), Tizanidine (2mgx2)
9	50	F	13	Alpha 2	Pz	4 Hz	Gabapentin (300 × 2), Duloxetine (60 mg), Lormetazepam (2 mg), Zolpidem (50 mg), Sirdalud (2 mg), Tapendatol (50 mgx2)
10	50	F	16	Theta	F4	30 Hz	Pregabalin (100 mg), Duloxetine 60 mg
11	65	F	9	Delta	C3	30 Hz	–
12	53	F	11	Theta	Cp5	30 Hz	–
13	57	F	13	Theta	Cp5	30 Hz	Duloxetine (60 mg); Pregabalin (75 mg)
14	52	F	8	Beta	C3	4 Hz	–
15	53	M	17	Beta	Pz	4 Hz	–

### EEG

**Fig. 3:**
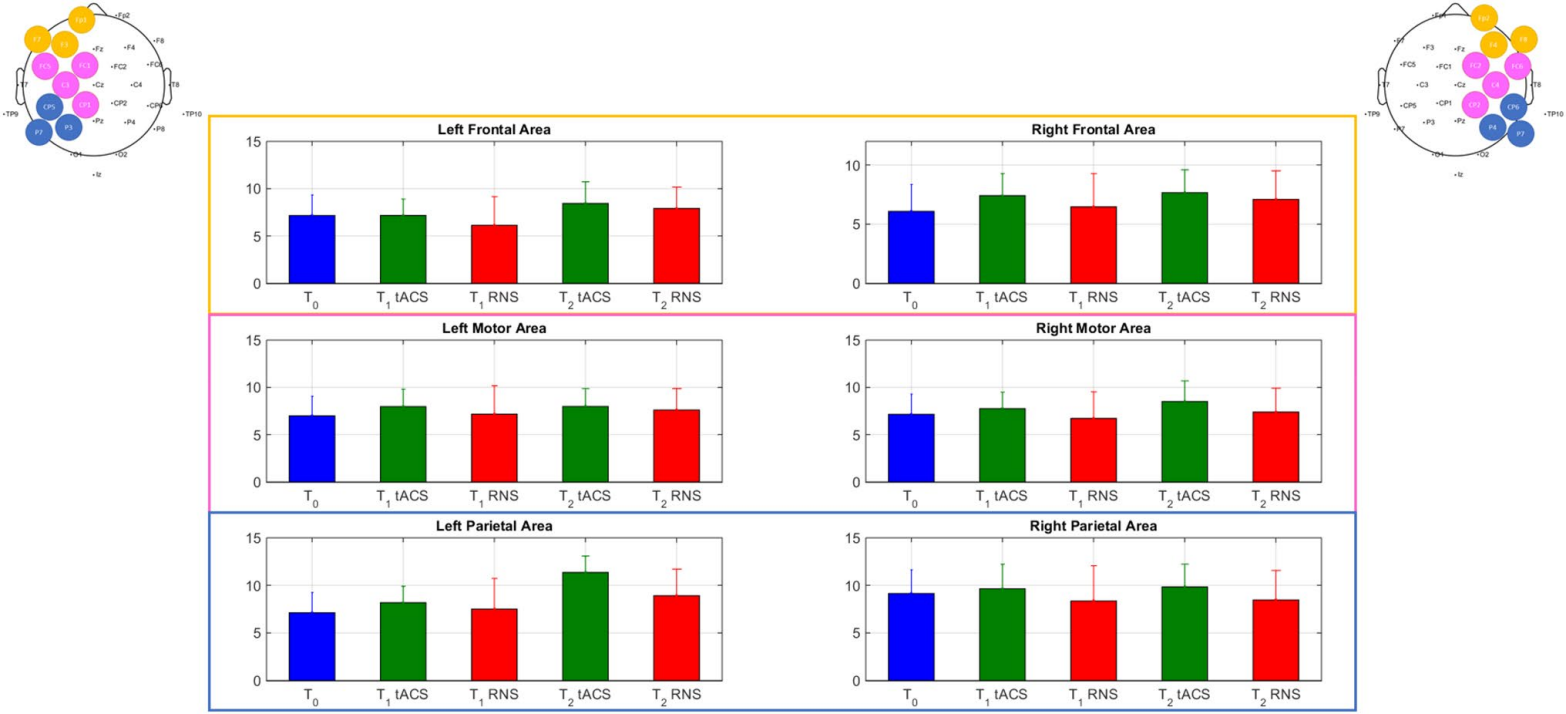
Group results. Median relative power and standard error in alpha1 range at each evaluation time point (*T*_0_, *T*_1_ tACS, *T*_1_ RNS, *T*_2_ tACS, *T*_2_ RNS). Region of interests are identified based on electrodes location (mean of relative power): left frontal area —i.e., Fp1, F7, F3; right frontal area—i.e., Fp2, F8, F4; Left motor area—i.e., FC1, FC5, C3, Cp1; right Motor Area—i.e., FC2, FC6, C4, Cp2; left Parietal Area—i.e., CP5, P7, P3; Right parietal area—i.e., CP6, P8, P4

The open-eyes resting-state EEG confirmed low rhythm prevalence over fronto-central cortical regions (11/15 participants) [13, 15]. Alpha1 power increased at *T*_1_ (*p* = 0.024, CI (− 1.89, − 0.13)) after beta-tACS compared to RNS over bilateral M1 (Fig. 3). Alpha 2 showed a non-significant increase between *T*_1_ and *T*_2_ both after tACS and RNS over bilateral M1.

### Clinical assessment scales

VAS scores significantly decreased in 9 cases out of 14 from *T*_0_ to *T*_1_ (*p* 0.010, CI (0.5, 4.0)) after tACS. This effect was no longer seen at *T*_2_, where just 4 subjects out of 14 reported a reduced pain perception compared to *T*_1_.

After RNS, no significant improvement was seen between *T*_0_ and *T*_1_ as just in 7 cases out of 14 we observed a reduction in VAS scores (*p* = 0.062) as well as between *T*_1_ and *T*_2_ where just 4 subjects reported pain symptoms reduction (*p* = 0.757).

Several items of the Short Form 36-item Health Survey (SF-36) improved after both tACS and RNS. See the online resources (Tables T2, T4) for significant p-values and median scores related to each subitem.

### Neuropsychological and psychopathological assessment

1. Psychopathological self-assessment: the State–Trait Anxiety Inventory, assessing baseline state and trait anxiety levels, shows low trait anxiety in our sample at baseline (mean $\pm$ standard deviation = 37.26 $\pm$ 7.63; median = 38). Depressive symptoms, assessed with the BDI-II, decreased in the tACS group between *T*_0_ and *T*_1_ (*p* < 0.001, CI (4.0, 12.0)), and *T*_0_ and *T*_2_ (*p* = 0.006, CI (5.0, 13.0)), as well as in the RNS group between *T*_0_ and *T*_1_ (*p* = 0.008, CI (1.0, 10.5)). The BSI global severity index score, a measure of the current level of perceived symptomatology, significantly decreased after both tACS and RNS between *T*_0_ and *T*_1_ (respectively, *p* = 0.042, CI (0.01, 0.11), and *p* = 0.027, CI (0.02, 0.23)). Several subitems of the BSI improved after both tACS and RNS. Tables T3 and T5 in the online resources, respectively, report the p-values and the median scores for each subitem.
2. Self-reported cognitive assessment: the skill loss subitem of the Patient-Reported Outcomes in Cognitive Impairment (PROCOG-P) improved after tACS at *T*_0_–*T*_2_ (*p* = 0.024, CI (0.05, 0.59)). The performance in the Everyday memory questionnaire (EMR.Q) resulted significantly improved in the group receiving the tACS compared to the RNS at *T*_1_ (*p* = 0.012, CI (1.5, 16.5)).
3. Neuropsychological assessment: among neuropsychological tests, a significant improvement is observed in the MoCa scores after tACS at *T*_0_–*T*_1_ (*p* = 0.048, CI (− 4.5, 0.0)) and at *T*_0_–*T*_2_ (*p* = 0.009, CI (− 4.5, − 0.5)). In the Trail Making Test A (TMT-A), we observed a significant increased speed in the RNS group between *T*_0_ and *T*_2_ (*p* = 0.035, CI (0.0, 15.0)). The TMT-B time significantly decreased in the tACS group between *T*_0_ and *T*_2_ (*p* = 0.034, CI (0.0, 37.5)). Total Phonemic Fluency scores increased in the group receiving tACS between *T*_0_ and *T*_2_ (*p* = 0.013, CI (− 10.0, − 1.0)) and errors decreased (*p* = 0.025, CI (0.0, 2.0)). In the RNS group, an increase was observed between *T*_1_ and *T*_2_ (*p* = 0.006, CI (− 9.0, − 1.5)). In the HVLT-R, the tACS group improved significantly both at *T*_0_–*T*_2_ and *T*_1_–*T*_2_ (respectively, *p* = 0.008, CI (− 9.0, − 1.0), and *p* = 0.009, CI (− 5.5, − 1.0)); the RNS group improved between *T*_0_ and *T*_2_ (*p* = 0.012, CI (− 14.5, − 2.0)). The Rey–Osterrieth Complex Figure time score improved in the tACS group between *T*_0_ and *T*_1_ (*p* = 0.025, CI (7.0, 49.0)), and *T*_0_ and *T*_2_ (*p* = 0.049, CI (0.0, 42.0)), and between *T*_0_ and *T*_2_ after RNS (*p* = 0.037, CI (3.5, 69.0)).

In the online resources (Table T6) are reported the median scores of each administered test.

## Discussion

Our data confirm cognitive and EEG activity abnormalities in a sample of people with FMS. This neurophysiological finding informed the choice of the neurostimulation paradigm: tACS combined with an ad hoc physical program was effective in shifting EEG frequencies, reducing pain, and improving neuropsychological and psychopathological tests.

Slow rhythm prevalence in fronto-central cortices are a hallmark of FMS [16]: fast tACS aimed to interact with these abnormal brain oscillations and shift them towards more physiological frequencies [18, 25]. Eleven out of fifteen participants of the initial sample showed theta rhythm prevalence in frontal regions and/or sensorimotor areas. As hypothesized, tailored tACS normalized EEG activity [48]. Although beta-tACS decreases the prevalent theta power in our sample, the induced shift was towards slightly faster (alpha1 or alpha2) bands and not towards beta band. This observation may be explained with the complexity of the targeted pain circuit, the pain matrix [49]. Neuroimaging and neurophysiological studies demonstrated that nociceptive stimulations activate large brain network comprising somatosensory, insular, cingulate areas, and with a temporal delay frontal and parietal areas [50]. FMS pain-related symptomatology is believed to be associated with neuroplastic changes in this network [51]. Taking this into account, we argue that focal tACS stimulation is less likely to radically impact on a large cortical/subcortical network, like the one represented by the pain matrix. Indeed, tACS efficacy in EEG activity normalization was observed in the treatment of other thalamo-cortical dysrhythmias such as in Parkinson’s disease, in which the closely circumscribed thalamo-cortico-basal circuit was targeted [18].

We found the primary motor cortex (M1) and sensorimotor areas to emerge as the main targets for the neurostimulation based on the topography of EEG abnormalities. Although M1 is not directly part of the pain matrix, previous studies proved its modulatory role in other chronic pain syndromes [51]. M1 stimulation appears to activate phasic and rapid activity of lateral thalamic nuclei, which in turn activate a cascade of events in the medial thalamus, anterior cingulate/orbitofrontal cortex and periaqueductal gray matter.

Thus, we argue that tACS delivered over M1/sensorimotor cortices may have a modulatory effect on the pain matrix and consequently reduce pain, as proved by reduced VAS score. The absence of significant improvements at *T*_2_ confirms previous reports [52, 53] on the lack of long-lasting effects of NIBS: our experimental paradigm was able to modulate pain during the experiment itself and immediately after, but the pain reduction faded after 4 weeks. This observation calls for the development of portable NIBS devices which may be used for home therapy. In addition, the lack of effects after RNS can be considered a proof as well of the benefit induced by the real stimulation and physiotherapy treatment.

Physiotherapy may be a co-factor in pain reduction; however, as only the tACS group reported a significant decrease of VAS, an effect of stimulation per se accounting for this outcome is highly likely. TACS may have acted as a primer for the motor cortex, increasing the potential excitability of the underlying cortex and amplifying the ensuing physiological activation obtained through motion.

While the results of tACS on pain suggest a determinant role of neurostimulation, the overall positive effect of physical activity on health perception is well known and observed also in our cohort.

A general health increased is observed in both groups (tACS and RNS), as assessed by SF-36 scores of functionality, energy, social activity and pain subitems.

Similraly, physical activity may have affected also BSI and BDI-II. BSI assesses somatization, obsessive compulsive tendencies and anxiety; scores improved in both groups between *T*_0_ and *T*_1_. BDI-II assesses depressive symptoms; it decreased significantly in short and medium term (*T*_0_–*T*_1_ and *T*_1_–*T*_2_) in the tACS group. It is unclear if this improvement results from a primary effect of the stimulation and the physiotherapy, both previously demonstrated to be effective in depressive symptoms amelioration [54, 55] or if it is a consequence of pain reduction per se. Pain and depression are strictly linked; thus, a reduction of one can lead to beneficial effects on the other. However, depressive symptoms seem to ameliorate also in the RNS group in the short term (*T*_1_), which did not show a significant reduction in VAS scores. In this case, the positive effect of physiotherapy may have played a determinant role.

The PROCOG-P skill loss subitem as well as the EMR.Q improvement in the tACS group can be interpreted as a consequence of the stimulation, which by increasing alpha1 band prevalence, may have boost cognitive performances and the relative perception of self-cognitive abilities. However, pain and depression reduction may have played a role by influencing subjects’ general attitude on their capacities. This underlies the need to test cognitive abilities also with neuropsychological standardized test, to disentangle subjective and objective measures of cognitive functioning.

A positive correlation between theta power increase and cognitive deficit in healthy adults is reported [56]; whereas, high power of alpha rhythm is positively correlated with memory and attention performances [57]. TACS stimulation, increasing alpha1 band prevalence, may explain the observed improvements in the MoCA scores, which comprises attention, short-term memory and working memory tasks.

The same can be assumed for TMT-B performance assessing divided attention and set-shifting, whose improvement is observed only in tACS condition. On the contrary, TMT-A performance speeds up only in the RNS group. We argue that this task, assessing mainly visual searching abilities, could have been less influenced by the protocol as less involved in FMS symptomatology compared to the other domains tested.

The other cognitive tasks comprising the phonemic fluency test, the HVLT-R and the time copy component of the Rey Complex Figure test, improved both after tACS and RNS stimulation. Faster improvement in performances were observed at *T*_1_ after tACS, compared to the RNS later improvement (*T*_2_). We argue that tACS combined with physical activity is likely to speed up the process of cognitive performance improvement in FMS, which can anyway be triggered by physical rehabilitation. Indeed, many studies highlight the modulation effect of physical activity on cognitive functioning and general wellbeing [58].

By delivering tailored tACS associated with ad hoc rehabilitative intervention, we succeeded in reducing the main concerns reported by people suffering FMS [59]: pain symptoms and cognitive dysfunctions, including both self-reported measures of perceived impairment and neuropsychological tests performance.

The improvements observed in the group receiving RNS combined with physiotherapy can be explained in light of the multiple-level beneficial impact of physical activity on clinical and cognitive symptoms. However, tACS group showed more pervasive and faster symptoms reduction, pointing out stimulation efficacy.

## Limitations

The main limitation is the small sample size. Nevertheless, the sample homogeneity concerning age, sex and education level, adds value to results reliability.

It is pointed out that, considering the dimension of the sample size and the exploratory nature of the study, no corrections for multiple comparisons were performed in hypothesis testing. Future studies should replicate the validity of this treatment approach on a larger sample.

A potential bias could be concomitant drug therapy. Evidence to date suggests interaction effects between drugs with psychotropic effects and neurostimulation techniques [60] Even if not such interaction is clearly reported for tACS, we controlled for possible confounding effects by keeping participants’ pharmacological therapy unchanged during the whole protocol.

## Conclusion

These data provide evidence of the efficacy of combining personalized tACS and physiotherapy in the treatment of pain, cognitive symptoms and subclinical psychopathology of FMS. Even if the involved mechanisms are still not completely understood, tACS over the sensorimotor cortex coupled with physiotherapy seem to be a promising approach in treating this complex syndrome.

### Electronic supplementary material

Below is the link to the electronic supplementary material.

Supplementary material 1 (docx 37 kb)

Supplementary material 1 (docx 25 kb)

## Appendix A. Neuropsychological testing

### Clinical and Psychopathological assessment

The beck depression inventory-II (BDI-II) [35] was used to assess depression. It consists of a 21-item questionnaire yielding a composite score of self-reported symptom severity. Standard cut-off scores are: 0–9 = minimal depression, 10–18 = mild depression, 19–29 = moderate depression, and 30–63 = severe depression.

Brief symptom inventory (BSI) [36] is a self-reported questionnaire designed to identify relevant psychopathological symptoms experienced during the last week. The questionnaire is composed by 53 items concerning nine symptom dimensions: Somatization, Obsession–Compulsion, Interpersonal Sensitivity, Depression, Anxiety, Hostility, Phobic Anxiety, Paranoid ideation, and Psychoticism. The questionnaire provides also three global indices of distress: Global Severity Index (a measure of current level of symptomatology), Positive Symptom Distress Index (a measure of the intensities of symptoms), and Positive Symptom Total (a measure of total number of experienced symptoms). GSI T scores > / = 63 are considered clinically significant, also all cases in which two of the subscales scores are 63 or greater.

The state–trait anxiety inventory [37] is a 40-item self-report questionnaire assessing state and trait anxiety levels. Scores range from 20 to 80, with higher scores indicating higher levels of anxiety severity.

### Cognitive self-assessment

Patient-reported outcomes in cognitive impairment (PROCOG-P) [38] is a 55-item self-administered questionnaire designed to measure a range of patient-reported symptoms and their impact in patient’s daily life during the last two weeks. The instrument is designed to detect the patient’s perspective on his/her cognitive impairment and impact. Items are rated on a five-point Likert scale and the questionnaire includes seven subscales whose scoring is calculated as the mean value of items belonging to each specific subscale (range 0–4).

The PROCOG-P subscales are: affect, skill loss, semantic memory, short-term memory, cognitive functioning, long-term memory and social impact. The subscales give extensive description of the patient experience of his/her cognitive impairment and allow us to assess separately different memory-related symptoms and the emotional impact of symptoms and repetitive behaviors. A total score is the sum of all items (range 0–220). Higher scores indicate both greater impact and severity of cognitive impairment.

Everyday memory questionnaire revised (EMQ-R) [39] is a subjective measure of memory deficiency in everyday life during last month. Total score is the sum of all 13 items, ranged from 0 to 41, with a mean total of 9.75 (SD 8.6), and it is considered as a good measure of change. Higher the score, worse subjective memory functioning.

### Neuropsychological assessment

The montréal cognitive assessment (MoCA) [61] is a 30-point brief cognitive screening scale with short time of administration. MoCA is one of the most commonly used tools in clinics assessing general cognitive functioning. It measures a broad spectrum of cognitive abilities that are relevant to several disorders involving CNS [61]. In particular, MoCA was developed to explore frontal cognitive domains (i.e., attention, executive functions, and conceptual thinking), all domains usually already affected in the early stages of these disorders. Authors identified scores less than 26 as a good cut-off for detecting cognitive impairment.

The Rey–Osterrieth complex figure test [41] is a measure of visuo-spatial constructional abilities and visuo-graphic memory, but also cognitive planning, organizational strategies and executive functions. The task is composed of two parts, direct copying (assessing perception and visuo-spatial construction) and delayed reproduction (assessing implicit visuo-spatial memory). We used the three minutes (short) delay to assess visual memory. Given that repeated administrations of the ROCF resulted in significant improvements of performance, we used alternative forms: The Modified Taylor Complex Figure, and two out of the four complex figures devised for repeated assessments by the Medical College of Georgia Neurology group [62].

The digit symbol-coding (from the Wechsler Adult Intelligence Scale 4th edition [42]), is a measure of grapho-motor working memory and speed of processing. It consists of digit–symbol pairs followed by a list of digits. Participants must write the corresponding symbol under each digit (ranged from one to nine) as fast as possible in a limited time interval (120″). Digit Symbol appears to be relatively unaffected by intelligence, memory, or learning. Motor persistence, sustained attention, response speed, visuomotor coordination, all have some role in digit symbol performance, which is also affected by education, gender and age. No practice effects appeared after repeated administering [62].

The hopkins verbal learning test-revised [43] is a test that assesses verbal learning and memory. The test consists of three trials of free recall of a 12-item composed of four words belonging to three different semantic categories. The authors provide six parallel forms leading to equivalent results in the normal population. We used three lists (N 1, 5, and 6) and considered only the free recall as outcome measure.

The trail making tests A and B [44] measure attentional speed, sequencing, visual search and mental flexibility. Part A (TMT-A) assesses motor speed, part B (TMT-B) assesses complex divided attention and set-shifting, difference between B and A (i.e., B/A ratio) gives a measure of cognitive shifting cost and allows us to control for motor impairment. Practice effect is under discussion, especially in the case of short time interval, and we used three parallel forms [63].

The phonemic verbal fluency task [45] requires patients to freely generate as many words as possible that begin with a specific letter (phonemes) in 60 s. The task requires patients to retrieve words of their language and to access their verbal lexicon, focus on the task, select only words following specific rules and avoid repetitions and words that start with phonemes close to the target one. It is therefore considered dependent on executive control, beyond the involvement of verbal abilities. The outcome consists in the total correct words produced through three letters. In the literature different letter combinations are available to longitudinal studies.
