# Supplementary material for: Beyond physiotherapy and pharmacological treatment for fibromyalgia syndrome: tailored tACS as a new therapeutic tool
Source: Eur Arch Psychiatry Clin Neurosci. 2020 Nov 25;271(1):199–210. doi: 10.1007/s00406-020-01214-y (PMC7867558; doi:10.1007/s00406-020-01214-y)
Supplement: Supplementary file 1 — Supplementary material 1 (docx 37 kb) [file 406_2020_1214_MOESM1_ESM.docx]

Figure F1

Study diagram flow

**Enrolment:**

24 subjects with FMS diagnosis assessed for eligibility

**7 Subjects excluded (not meeting inclusion criteria):**

2 for contraindications to stimulation

3 for comorbidity

2 for recent drug regimen changes

**Allocation:**

17 subjects randomized

**2 Dropouts before 1^st^ arm**

**start:**

1 for personal reasons

1 for drug regimen changes

**1^st^ Study Arm:**

15 subjects

**4 Dropouts before 2^nd^ arm**

**start:**

2 for personal reasons

1 drug regimen changes

1 stroke

**2^nd^ Study Arm:**

11 subjects

**Article Title:** “*Beyond physiotherapy and pharmacological treatment for Fibromyalgia syndrome: tailored tACS as a new therapeutic tool”*

**Authors:** Laura Bernardi*, Margherita Bertuccelli*, Emanuela Formaggio, Maria Rubega, Gerardo Bosco, Elena Tenconi, Manuela Cattelan, Stefano Masiero, Alessandra Del Felice

**Journal:** [European Archives of Psychiatry and Clinical Neuroscience](https://link.springer.com/journal/406)

**Corresponding author:**

Margherita Bertuccelli, MSc

NEUROMOVE-Rehab, Department of Neuroscience, University of Padova, Padova, Italy.

Department of Neuroscience, University of Padova

Via Giustiniani, 3, 35128 Padova, Italy

e-mail: [margherita.bertuccelli@phd.unipd.it](mailto:margherita.bertuccelli@phd.unipd.it)
