# Supplementary material for: Beyond physiotherapy and pharmacological treatment for fibromyalgia syndrome: tailored tACS as a new therapeutic tool
Source: Eur Arch Psychiatry Clin Neurosci. 2020 Nov 25;271(1):199–210. doi: 10.1007/s00406-020-01214-y (PMC7867558; doi:10.1007/s00406-020-01214-y)
Supplement: Supplementary file 2 — Supplementary material 1 (docx 25 kb) [file 406_2020_1214_MOESM2_ESM.docx]

**Tables**

Table T1

Physiotherapy program

| **Activities** | **Basic exercises** |
| --- | --- |
| **Relaxion exercises (15 min)** | Breathing exercises to promote chest expansion (diaphragmatic and costal breathing).  Intersegmental coordination exercises Muscle tension awareness exercises  Right and Left asymmetries awareness exercises |
| **Active joint mobilization (10 min)** | Exercises for upper and lower limbs lying supine position, on the side, on all fours, sitting, standing  Exercises to release shoulder and pelvic girdle Pelvic anteversion and retroversion movements Exercises for cervical spine in sitting position (flexion-extension, lateral bending, rotation) Exercises for the trunk sitting and standing (flexion and rotation) The bridge exercise to tone the muscles of the anterior abdominal wall, glutei, quadriceps and hamstring |
| **Aerobic Training (20 min)** | Treadmill Cycle ergometer Exercise bike  Elliptical trainer |
| **Stretching exercises (15 min)** | Exercises to stretch the muscles of the posterior kinematic chain, the adductor muscle of the hip, the hip extra rotator muscle and lumbar muscles (in the supine position, each knee, in turn, is brought to the chest) |

Table T2

SF-36 subitems significant p-values and confidence intervals

| **TEST** | **SUBITEM** | **tACS** | | | **RNS** | | |
| --- | --- | --- | --- | --- | --- | --- | --- |
|  |  | T0 vs. T1 | T1 vs. T2 | T0 vs. T2 | T0 vs. T1 | T1 vs. T2 | T0 vs. T2 |
| SF-36 | Physical activity/functionality | < .001  (-275, -75) |  | .024  (-375, -50) |  |  | .023  (-325, -100) |
|  | Limitations due to physical health |  |  | .017  (-400, -100) |  |  | .008  (-350, -200) |
|  | Energy/Fatigue |  | .014  (-110, -10) | .010  (-190, -30) |  |  | .037  (-110, -20) |
|  | Mental health | .027  (-90, 0) |  |  | .018  (-130, -10) |  |  |
|  | Social activities | .002  (-62.5, -25) |  | .016  (-150, 62.5) | .002  (-75, -25) |  | < .001  (-75, -25) |
|  | Pain | .004  (-87.5, -20) |  | .012  (-62.5, -20) | .004  (-80, -25) |  | .010  (-92.5, -20) |

Table T3

BSI subitems significant p-values and confidence intervals

| **TEST** | **SUBITEM** | **tACS** | | | **RNS** | | |
| --- | --- | --- | --- | --- | --- | --- | --- |
|  |  | T0 vs. T1 | T1 vs. T2 | T0 vs. T2 | T0 vs. T1 | T1 vs. T2 | T0 vs. T2 |
| BSI | Somatization index | .012  (0.29, 1.79) |  | .021  (0.15, 1.65) | .008  (0.43, 0.53) |  | .010  (0.36, 1.86) |
|  | Obsessive compulsive index |  |  | .035  (0.09, 1.00) |  |  | .016  (0.09, 1.92) |
|  | Interpersonal sensitivity |  |  | .005  (0.13, 1.25) |  |  |  |
|  | Anxiety index |  |  |  | .012  (0.17, 1.00) |  |  |
|  | The Global Severity Index | .042  (0.01, 0.11) |  |  | .027  (0.02, 0.23) |  |  |
|  | The Positive Symptom Distress Index | .015  (0.10, 0.72) |  |  | .004  (0.35, 1.16) | .012  (-0.65,-0.09) |  |
|  | Total positive symptom index |  | .025  (0.50, 6.0) | .015  (3.0, 15.0) |  |  | .032  (1.0, 17.0) |

Table T4

SF-36 subitems median values

| **TEST** | **SUBITEM** | **tACS** | | | **RNS** | | |
| --- | --- | --- | --- | --- | --- | --- | --- |
|  |  | T0 | T1 | T2 | T0 | T1 | T2 |
| SF-36 | Physical activity/functionality | 500 | 775 | 800 | 500 | 625 | 800 |
|  | Limitations due to physical health | 0 | 150 | 400 | 0 | 150 | 350 |
|  | Energy/Fatigue | 160 | 180 | 220 | 160 | 160 | 180 |
|  | Mental health | 330 | 360 | 380 | 330 | 380 | 360 |
|  | Social activities | 125 | 150 | 150 | 125 | 137.5 | 162.5 |
|  | Pain | 70 | 90 | 100 | 70 | 90 | 120 |

Table T5

BSI subitems median values

| **TEST** | **SUBITEM** | **tACS** | | | **RNS** | | |
| --- | --- | --- | --- | --- | --- | --- | --- |
|  |  | T0 | T1 | T2 | T0 | T1 | T2 |
| BSI | Somatization index | 1.93 | 0.79 | 0.43 | 1.93 | 0.71 | 1.00 |
|  | Obsessive compulsive index | 1.00 | 0.84 | 0.50 | 1.00 | 0.59 | 0.50 |
|  | Interpersonal sensitivity | 0.75 | 0.25 | 0.00 | 0.75 | 0.38 | 0.00 |
|  | Anxiety index | 0.83 | 0.50 | 0.33 | 0.83 | 0.42 | 0.42 |
|  | The Global Severity Index | 0.21 | 0.12 | 0.08 | 0.21 | 0.08 | 0.12 |
|  | The Positive Symptom Distress Index | 1.91 | 1.33 | 1.33 | 1.91 | 1.25 | 1.36 |
|  | Total positive symptom index | 26.5 | 15.0 | 12.0 | 26.5 | 13.5 | 12.0 |

Table T6

Median values of the tests

| **TEST** | **Baseline** | **tACS** | | **RNS** | |
| --- | --- | --- | --- | --- | --- |
|  | **T0** | **T1** | **T2** | **T1** | **T2** |
| VAS | 7.0 | 5.5 | 5.5 | 5.5 | 4.0 |
| Beck Depression Inventory-II (BDI-II) | 12.0 | 6.0 | 5.0 | 5.0 | 6.0 |
| State-Trait Anxiety Inventory | 38.0 | 36.0 | 32.0 | 36.5 | 34.0 |
| Patient-Reported Outcomes in Cognitive Impairment (PROCOG-P) | 46.0 | 34.0 | 35.0 | 39.5 | 41.5 |
| Everyday Memory Questionnaire Revised (EMQ-R) | 10.0 | 11.0 | 11.0 | 5.0 | 10.5 |
| The Montréal Cognitive Assessment (MoCA) | 25.0 | 27.5 | 28.0 | 27.5 | 26.0 |
| The Rey-Osterrieth Complex Figure Test (Time) | 100 | 89.0 | 86.0 | 90.0 | 73.0 |
| The Digit Symbol-Coding | 46.0 | 47.5 | 47.0 | 51.5 | 50.0 |
| The Hopkins Verbal Learning Test-Revised | 21.0 | 23.0 | 26.0 | 26.0 | 27.0 |
| Trail Making Test A | 40.0 | 32.5 | 31.5 | 30.0 | 33.0 |
| Trail Making Test B | 77.0 | 55.0 | 60.5 | 52.5 | 60.0 |
| Phonemic Verbal Fluency task (TOT) | 37.0 | 41.0 | 43.0 | 34.5 | 39.0 |
| Phonemic Verbal Fluency task (errors) | 1.0 | 0.5 | 0.0 | 0.0 | 1.0 |

**Article Title:** “*Beyond physiotherapy and pharmacological treatment for Fibromyalgia syndrome: tailored tACS as a new therapeutic tool”*

**Authors:** Laura Bernardi*, Margherita Bertuccelli*, Emanuela Formaggio, Maria Rubega, Gerardo Bosco, Elena Tenconi, Manuela Cattelan, Stefano Masiero, Alessandra Del Felice

**Journal:** [European Archives of Psychiatry and Clinical Neuroscience](https://link.springer.com/journal/406)

**Corresponding author:**

Margherita Bertuccelli, MSc

NEUROMOVE-Rehab, Department of Neuroscience, University of Padova, Padova, Italy.

Department of Neuroscience, University of Padova

Via Giustiniani, 3, 35128 Padova, Italy

e-mail: [margherita.bertuccelli@phd.unipd.it](mailto:margherita.bertuccelli@phd.unipd.it)
